# The Xenopus Oocyte: A Tool for Membrane Biology

**DOI:** 10.3390/membranes13100831

**Published:** 2023-10-15

**Authors:** Agenor Limon, César Mattei

**Affiliations:** 1 Mitchell Center for Neurodegenerative Diseases, Department of Neurology, The University of Texas Medical Branch, Galveston, TX 77555, USA; aglimonr@utmb.edu; 2 Univ Angers, CarMe, Unité MITOVASC, UMR CNRS 6015, INSERM U1083, SFR ICAT, 49000 Angers, France

The Xenopus is a special study model in experimental research. In embryology, it has been used to understand the harmonious development of a complex organism from its fertilized cell. In toxicology, it is the model of choice for observing the deleterious effects of endocrine disruptors. In pharmacology, female oocytes have been used for over forty years to express genes of interest. In fact, the membrane of Xenopus oocytes is relatively poor in membrane receptors and ion channels. Furthermore, these cells are relatively large (1–2 mm), making it easy to express proteins by injecting DNA or RNA, or by transplanting membranes using a xenograft protocol. These techniques have enabled scientists to characterize receptors and transporters outside their physiological context.

Our Special Issue, entitled “The Xenopus Oocyte: A Tool for Membrane Biology” in the journal Membranes, aimed to give this cell, which is widely utilized by researchers working on membrane proteins, the place it deserves in our time. Most of the articles that are published in this issue use the oocyte as an expression system, i.e., a cell in which we express or overexpress a protein whose normal or altered functioning we wish to understand. Indeed, the heterologous expression of membrane receptors is indispensable for understanding the changes, particularly electrophysiological, that are induced by mutations in the corresponding gene or through their targeting with pharmacologically active molecules.

The expression of various receptors and ion channels therefore constitutes most of the papers published in this Special Issue. Using a classical electrophysiology approach based on two-electrode voltage clamp recording, Bertaud et al. [1] compared the effect of several widely used insecticides on human and insect GABA receptors. The use of the Xenopus oocyte is underlined by the pharmacological differences that exist between these GABA receptors in their differential sensitivity to insecticides. Rousset et al. [2] have adapted the membrane transplantation method that was developed in the 1990s by Miledi [3] to characterize the electrophysiological properties of voltage-gated Ca^2+^ channels from microtransplanted mouse tissues. This original method could eventually serve as a screening platform for channelopathies. The same approach has been used by Miller et al. [4] to characterize the currents that are linked to the activation of metabotropic glutamate receptors (mGluRs): the microtransplantation of synaptosome membranes from rat cortexes facilitates the study of their properties. In a detailed review, Ivorra et al. recall the principle of this membrane transplantation, as well as the advantages and disadvantages associated with this original methodology, initiated with Torpedo electroplates and subsequently extended to all types of mammalian and even invertebrate tissues [5,6,7,8].

Numerous membrane receptors can be expressed in the Xenopus oocyte to characterize their biophysical or pharmacological properties using the two-electrode voltage clamp technique. In a study by Isaev et al. [9], the antagonistic effect of methylene blue on K_ATP_ channels was demonstrated by measuring the intensity of a current induced by cromakalim. And Cav2.1 channel variants causing ataxic channelopathies revealed electrophysiological gains or losses of function in the study by Folacci et al. [10]; the molecular modelling of these mutations or of the associated neuronal excitability confirms the electrophysiological findings. Lummis and Dougherty explored the contribution of proline residues on α1 subunit glycinergic receptors to their sensitivity to glycine. By generating substitution point mutations, they demonstrated the functional importance of Pro residues [11]. Finally, Stein et al. used the oocyte to express tight junction proteins in a two-cell model [12]. Their data show that the activity of claudin proteins is largely pH-dependent.

However, far from being just an expression system, the Xenopus oocyte can also be used as a cell for the development of innovative membrane protein monitoring techniques. Thus, by coupling the measurement of voltage-gated Na^+^ and K^+^ currents with the optogenetic photosensitivity of channelrhodopsin, vom Dahl et al. [13] could observe and measure the characteristics of action potentials that were specific to excitable cells. Their work could lead to the development of a genuine pharmacological platform for testing therapeutic compounds and assessing the impact of genetic mutations on cell excitability. Some studies have focused on membrane targets that are endogenously expressed in the Xenopus oocyte. For example, Bernareggi et al. [14] investigated endogenous Cl^−^ currents (TMEM16A Ca^2+^-activated chloride channels). This study shows that a compound that is present in asbestos fibers, crocidolite, induces an indirect activation of these channels via an increase in intracellular Ca^2+^, shedding further light on the carcinogenic effects of asbestos.

In conclusion, the use of this very special cell, the Xenopus oocyte, has enabled numerous scientific teams to work on a considerable variety of receptors and transporters, approaching them from biophysical, pharmacological, biochemical, electrophysiological and toxicological angles, among others [15]. A number of studies that utilize this cell have been cited hundreds of thousands of times, highlighting its versatility and adaptability for the expression of any type of receptor [16]. It is a very convenient link between molecular expression work and its physiological or pathophysiological extensions. As Gamba points out, many fields of physiology, such as arterial blood pressure, neuronal excitability, mineral metabolism and cell volume regulation, are initiated using the Xenopus oocyte [17]. Easy to obtain and use, the Xenopus oocyte is a very practical system in the toolbox of scientists who wish to have a cellular model for expressing genes of interest.
